# Foreign

**Published:** 1841-11-20

**Authors:** 


### FOREIGN.

*On Hairs within the Eye.* By Dr. STELLING, of Cassel.—A blacksmith, a healthy man 34 years old, had the end of a wire driven with considerable force through the cornea, and to the depth of half an inch into the interior of the right eye. Next day he had acute inflammation of the whole globe, and a high degree of fever, which were reduced by antiphlogistic remedies. Within a few days the wound in the cornea completely closed, so as to leave but a small cicatrix; the chambers, which had been completely evacuated, were again filled, but a partial capsular cataract had formed. The sight, which had been entirely lost, improved considerably, and though at first it was double, (apparently from the axis of vision being altered by an adhesion of the iris,) it soon again became single, and slowly increased in clearness.

About six weeks after the injury, the attendant surgeon first discerned in the posterior chamber some hairs of different lengths, of which the author gives the following account, from an examination made about four years after the accident: The left eye is perfectly healthy; in the right the iris is discoloured as if from a former attack of chronic inflammation. The pupil of the latter eye is irregular, and there is a partial capsular cataract. One hair commences a line below the horizontal diameter of the pupil, and extends in an almost vertical direction (forming a slight arch, like that of the inner eyelashes) to the outer edge of the iris, where it comes upon the cornea. It is about five lines long, and pointed at both ends; it is light yellow at its lower extremity, and dark brown at the upper. The second hair begins near the vertical diameter of the pupil, on the lower and inner part of the pupillary margin of the iris, and extends obliquely upwards and outwards to the distance of a line and a half beyond the outer margin of the pupil. It is three lines long, and is pointed at one and probably at both ends; it is of a dark brown colour. A third hair lies just below that last described, and seems to come from the same root; it extends in the same direction, has a pointed extremity, is about three lines long, and of a yellowish-white colour. The appearance of two other hairs proceeds from some small black points attached to the capsule of the lens, and probably resulting from particles of uvea affixed to it, and adhering with fine filaments of fibrin passing from it to the iris. The small cicatrix is still visible in the middle of the cornea, and is marked by a black point, which is probably the consequence of a small portion of uvea having been left in the wound when the wire was drawn out directly after the accident. The eyelashes and eyebrows are of the same colour as the hairs within the eye. The patient suffers no pain in the affected organ, and its sight is not more impaired than by the opacity of the capsule of the lens.

The author discusses the question whether these hairs were really produced within the eye, as they sometimes are from growths on the conjunctiva, or whether they were carried into it by the wire, and had subsequently become adherent. He adopts the latter view, grounding his opinion chiefly on these facts: that there was no appearance of a growth from which they could originate; that they did not proceed from the same point; that they exactly resembled the man's eyelashes; that they did not grow; and that such a circumstance as the production of hair within the eye is not only unheard of, but most improbable. [On the other hand, it is surely scarcely less improbable that a wire should carry eyelashes through the cornea and leave no cixatrix on the lid, and that these should become adherent, and yet not remarked by a careful observer daily examining the eye for nearly six weeks.]—*Brit. and For. Med. Rev., from Holscher's Annalen.*

*On the Employment of Nitrate of Silver in White Swellings.* By M. JOBERT, Surgeon to the Hospital St. Louis.—By a series of accurate and conclusive observations, M. Jobert has shown that the best and most prompt means of overcoming articular pains in cases of white swelling, and to make the turgescence of the tissues disappear, consists in the external employment of an ointment of nitrate of silver. We have watched on fifteen patients the action of this remedy, in the wards of M. Jobert, and have been astonished at the prompt effects in long-continued and previously rebellious diseases. The treatment consisted in frictions on the diseased articulation with an ointment composed of thirty parts of lard to four of nitrate of silver. If the action of this be insufficient, M. Jobert uses eight or twelve parts of the salt to thirty parts of lard. These ointments, designated by the numbers 1, 2, 3, constitute the whole of the treatment. Twelve or fifteen hours after the first employment of the ointment, and generally after the second friction, an eruption of small accuminated pustules appears, presenting a black point in their centre, and surrounded at their circumference by a small rosy areola. The liquid contained in the vesicle at first resembling thick milk, and rapidly assuming a yellowish-white appearance, afterwards becoming true pus. Each friction is accompanied by pains which last three or four hours. About the second or third day the skin becomes of a violet colour, and smarts acutely. The frictions must then be suspended, and not renewed until the parts are calmed. We do not enter into further details, as a full memoir on the subject is promised.

[It is very extraordinary that such accurate pathologists as the French should so generally continue to class as "*tumeurs blanches*" the very different diseases to which the joints are subject. We are thus left in doubt whether the above cases were scrofulous enlargement of the articular extremities of the bones, ulceration of cartilage, disease of synovial membrane, or of parts external to the joint.]—*Ibid, from Bul. Gén. de Thérapeutique.*

*On the Contagiousness of the Pseudo-membranous Inflammation of the Mouth, Pharynx, Œsophagus, and Stomach.* By Dr. KESSLER, of Samter.—On the 1st of December, 1840, a robust woman, twenty years old, after exposure to cold, was seized with the usual signs of this disease, which continued till the thirteenth day, when the false membranes began to be thrown off, and the whole condition of the patient improved. After some days more she completely recovered.

Up to the fifth day of her illness she was nursed and watched by one of her relations, a woman named F.; she, however, became ill, and then a woman forty years old, and of rather a weak constitution, was attacked with the same disease, with a remarkable predominance of the nervous symptoms. The place of both these was then taken by an unmarried woman, S., twenty-seven years old, who had been reduced by a very laborious life, and she also fell ill with the same symptoms on the 9th of December.

At the same time another woman, G., who lived in the neighbourhood, and had often waited on the preceding, sickened with the same disease. In her it assumed many of the characters of typhus abdominalis, and she died on the 20th of December.

Another woman, twenty-eight years old, of a rather weak habit, and very excitable, now came to attend on F. and S., and waited on them. She became ill on the 15th of December with the same symptoms, and the disease in her took the same course as in them, the nervous symptoms being more than commonly predominant.

Her husband, a man fifty years old, who had drunk hard, and now attended steadily on his sick wife, fell ill with nearly the same symptoms on the 20th of December, and in him the disease deviated from its usual course into an attack of delirium tremens.

There were thus six cases of this membranous angina which in their mode of spreading evinced a contagious nature, or which, at least, seemed to spread only by contagion. The patients were of different ages and constitutions, occupied different positions, led different kinds of life, and, for the most part, inhabited different houses.—*Ibid, from Med. Zeitung.*

This adds another illustration of the contagiousness of this fortunately rather rare disease.

*On the cure of Ozæna.* By Dr. DETMOLD, of Hanover.—The author says he has never failed to cure ordinary ozæna (by which he means the chronic coryza accompanied by a stinking discharge from the nose and a flabby relaxed state of the Schneiderian membrane) by the use of an injection composed of one or two drachms of chloride of lime rubbed up in a glass mortar with thirteen ounces of decoction of rhatany root, and strained off after standing for half an hour. About half an ounce of this must be injected into the nose three or four times a day with a syringe whose point is sufficiently long to carry the fluid high up into the nasal passages. The use of the remedy should be accompanied by the occasional administration of purgatives. It is very beneficial also in cases of chronic otitis with offensive discharge from the ear.—*Ibid., from Holscher's Annalen*, 1840. Bd. v. Hft. i.

*M. Ricord's New Urethro-plastic Operation*, By M. HELOT.—F. J. M., a shoemaker, twenty-six years old, came under the care of M. Ricord on the 16th of June, 1840. He said that when seven years old he took it into his head to tie up his penis with a thread, and after tying it tightly a little way in front of the scrotum, there followed on the next day a considerable swelling of the adjacent parts, in the midst of which the thread disappeared, cutting its way through the skin. To the swelling and division of the integuments, which cicatrized almost as fast as they were divided, there was added a retention of urine, which lasted, the patient says, fourteen days. At the end of this time the urethra was divided by the thread, and a large quantity of urine came away; after this severe symptoms ensued, which, however, in six weeks entirely disappeared, leaving only a kind of constricted cicatrix around the whole circumference of the penis, with the two opposite ends of the divided urethra exposed. The patient was also affected with phymosis.

From this time, says the report, the patient's infirmity has scarcely troubled him. The emission of urine takes place in him as in those affected with hypospadias; during erections, which are complete, the penis remains perfectly straight, though the glans does not seem to participate in the erection so much as it should. Neither is this portion of the organ the seat of any of the sensations which are peculiar to it in its perfect state,

On the 1st of January this man was attacked by gonorrhœa eight days after connexion. The disease commenced in the posterior portion of the urethra, and it was not till four days afterwards that (the two orifices being in contact) the anterior part of the canal was affected. Then the discharge went on at once through the posterior aperture and both the anterior ones. When the patient came into the hospital the discharge was abundant and very purulent; the posterior or vesical portion of the urethra was the seat of pain in the passage of the urine; the other part was only a little painful on pressure.

The acute symptoms having yielded to reducing treatment, a daily dose of twenty-four grains of powdered cubebs were ordered. After a few days the discharge had considerably diminished in the posterior part of the urethra, but in the anterior it was unaffected; after six days' use of the cubebs it had entirely ceased in the former situation, but in the latter it still continued.

During this time the two portions of the urethra had been carefully kept from contact; but on now permitting them to come together the posterior part which had been cured was again infected by the anterior, and had again to be treated by cubebs. The anterior part was at the same time treated and cured by injections of nitrate of silver.

After this it was proposed to endeavour to cure the organic defect of his urethra by a plan somewhat similar to that which M. Segalas had once successfully employed. The patient was (on the 3d of November) placed as for lithotomy. A large catheter having been introduced by the opening of the posterior part of the canal into the bladder, an incision, commencing immediately behind the situation of the bulb, was made in the middle line of the perineum and into the membranous portion of the urethra, through which, with some difficulty, a female catheter was then passed into the bladder. This being fixed, M. Ricord next operated for the phymosis, that he might have more yielding integuments behind the glans; and then having revivified the edges of the apertures in the urethra he brought them together (after all bleeding had ceased) and fixed them by sutures. A bougie had, before drawing the sutures, been passed through the two parts of the urethra down to the catheter in the membranous portion.

After the operation the patient was kept very quietly, with his lower limbs somewhat raised, and in the evening he was bled. Next day the urine had flowed freely through the catheter in the perineum; the wound was wet with urine, and its edges were swollen; on the third day the sutures came out; there was no union, and the urine escaped by the fistulous opening as it did before the operation. The edges of the fistula, however, preserved a transverse direction, and such a thickness as to lead one to hope that fresh trials at union might be more successful. On the 15th of November a considerable abscess which had formed in the perineum was opened at the scrotum, and discharged for some time pus and urine; but after it had healed fresh attempts were made to induce all the urine to pass through the opening purposely made in the perineum by introducing into it each day a larger catheter. This method was at last successful, and on the 19th of January a new union of the aperture was made by twisted sutures over a bougie introduced to a shorter distance beyond the aperture than the first had been. Three days after this operation the two external pins were removed, and the union of the parts near them was found complete. Next day the two others were removed, and there remained but a very small aperture through which a little urine and pus flowed. This was touched with nitrate of silver, and in a week (on the 1st of February) it had closed; another then appeared, which remained for some time, but was also ultimately closed.

On the 12th of February, three months and some days after the first operation, the perineal catheter was withdrawn, and a small one introduced through the whole urethra. Through this latter the urine now all passed, and the wound in the perineum rapidly healed. At the time of the report the patient was perfectly well; the penis had regained its form and discharged all its functions normally.—*Ibid, from Annales de la Chirurgie.* Mai, 1841.

---

*Application of the Subcutaneous Method to the Operation for Strangulated Hernia.* By M. Jules Guerin.—In this case the hernia was a congenital epiplocele which had been strangulated for three days. The usual means of reduction had been applied, and the tumour had become hard, engorged, and the seat of commencing inflammatory action. After division of the two rings and of the antero-superior wall of the inguinal canal, the reduction was immediately effected. The wound did not inflame, nor did the slightest febrile symptom follow. The patient was able to rise on the eighth day, taking care to wear a bandage.—*Ibid., from Gaz. Med. de Paris.*

---

*On softened, encysted Tubercles in the substance of the Uterus, as a cause of difficult Labour.* By Professor Osiander, of Göttingen.—The case here recorded differs from almost all others in which labour has been impeded by the pressure of tumours. Parturition was not impeded merely by their mechanical action, nor was the pelvic cavity contracted by their presence. They produced an injurious effect by paralyzing the action of the uterus and preventing the expansion of its fibres. The obstruction to labour thus caused was quite as great as if the pelvis had been contracted, and delivery could be effected only by mutilating the child and employing the blunt hook.

The patient was a woman, forty-five years old, who had suffered from scrofula in her infancy. She was a person of unhealthy aspect, had already miscarried twice, but had never given birth to a living child. When seen by Professor Osiander, she had been twenty-four hours, in labour, her strength was much exhausted, and she was very low spirited. The head of the child was felt to be very high up in the pelvis, and the membranes were still entire, though the os uteri was freely dilated.

After waiting for some hours, during part of which time the uterine action had been energetic, the head came somewhat lower down, and Professor Osiander ruptured the membranes. The head, however, remained above the brim of the pelvis, where it presented in the oblique diameter with the posterior fontanelle directed towards the right sacro-iliac synchondrosis. An attempt to bring down the head with the forceps was unsuccessful, and it was next sought to deliver the patient by turning. The hand of the operator could touch the ribs, but it was found impossible to reach the feet, for a sort of stricture in the middle of the uterus rendered all attempts to carry the hand as far as the abdomen, unavailing. Changing the position of the patient and placing her on her knees did not diminish the difficulties, for the contraction seemed to occupy alike all the walls of the uterus, and neither the right nor left hand could penetrate beyond it.

A second unsuccessful effort was made to deliver with the forceps; and then, after waiting for half an hour, Professor Osiander proceeded to perform craniotomy, and to extract the child by means of the blunt hook. This was not effected without great difficulty. It was necessary to introduce the hand, in order to remove the placenta, which was not adherent, but merely retained by an irregular contraction of the uterus. No serious hæmorrhage followed delivery, but the uterus never contracted properly, and the abdomen continued much distended. The patient lay in a listless condition, making no complaint of pain, but with a quick pulse and tumid abdomen which were thought to indicate the propriety of venesection. No relief followed its employment, the patient was soon afterwards attacked with vomiting, and died on the third day after delivery.

On a post-mortem examination, no traces of peritonitis were found; but the whole right side of the abdomen was occupied by the enormously large uterus. The substance of that organ was nearly three fingers thick, beset with hard swellings like eggs, of a somewhat oval form, and filled with a yellow caseiform matter resembling pus, the liquid parts of which had been absorbed. These large tubercles, about nine or ten in number, were invested with a fibrous envelope. They projected on the posterior and external surface of the uterus, so as to render it uneven. Many smaller bodies of the bigness of cherries were imbedded in the uterine parenchyma, and on a section being made of them were seen to be made up of concentric fibres; thus resembling in structure the ordinary fleshy tubercles of the uterus. With the exception of partial ossification of the left ovary, the above was the only morbid appearance of moment, and to this state of the uterus the difficulty experienced in introducing the hand must be exclusively attributed.—*Ibid., from Hannoversche Annalen.* v. Band. 15tes Heft.

---

*On the Oxidation of Gelatine.* By M. Persoz.—M. Persoz has determined that when gelatine is submitted to an oxidizing agent, it is susceptible of being transformed into hydrocyanic acid, ammonia, and carbonic acid, and a small quantity of one of the fat volatile and odoriferous acids, the existence of which was established by M. Chevreul. M. Persoz remarks that this fact leads to analytical researches to discover whether, among the products of normal or abnormal cutaneous secretions, we cannot discover ammonia, hydrocyanic acid, or some one of its derivatives, as those composed of cyanogen and formic acid. He thinks that in certain cases of suppuration, hydrocyanic acid may be formed, and states that M. Nonat has seen bandages and charpie tinted greenish-blue after having been for a long time in contact with the purulent matter of an abscess. This colouring may be attributed to Prussian-blue; but to verify and explain the fact, it will be necessary to observe the effect the pus of different wounds produces on pieces of linen impregnated with a salt of iron.—*Ibid., from Gaz. Med. de Paris.*

We have seen, in one case of chancres of very long standing in a broken constitution, a very intense blue tint given to all the dressings. The greenish-blue colour alluded to in the above article, is not of extremely rare occurrence in various forms of ulceration. It is occasionally seen in chronic ulcerations of the leg; but, in drawing inferences from such appearances, care should be observed to avoid the possibility of deception from the action of agents employed in the treatment of the sore, or in the preparation of the dressings.

In the venereal case just mentioned, nothing foreign to the suppuration was present locally during the time of treatment, except unbleached domestic muslin, the ordinary new patent lint, simple cerate, the basilicon ointment, and, occasionally, the lunar and the vegetable caustics. The latter was twice applied, but the blue tint—greenish at the edges of the spots—was not an occasional, but a constant feature of the case. This individual was twice bled during the treatment, and on both occasions the serum presented a milky hue, much deeper than that observed in the rice-water discharges of Asiatic cholera. The case occurred in the Pennsylvania Hospital, in 1821. The patient recovered. As the appearance may prove a diagnostic sign, we have thought it right to add this note. R. C.

---

*Intussusception and Separation of a part of the Ileum.* By Dr. Forcke, of Goslar.—A mechanic, forty-nine years old, had been in the campaigns of 1812–13–15, and from that time had very often suffered from colic, and other signs of abdominal disorder. In March, 1838, when first seen, he had not left his bed for five months; he was excessively emaciated, pale, and broken down by irritative fever; during all this time, also, he had suffered from costiveness, gripes, and vomiting. On examining the abdomen, a large, long, hard, and sensitive tumour was felt on the right side, which occupied the usual position of the cæcum and ascending colon. Various anti-spasmodic means produced but little effect on the symptoms from which he suffered. The only ease he received was derived from his bowels being opened, and from the use of enemata containing opium. The pains in the tumour and in the abdomen rising to a fearful height, the patient was ordered calomel and opium, and emollient poultices over the whole abdomen. These diminished the pain, and produced free evacuations, rest, and sleep; but the tumour remained unaltered. On the 7th of April, a severe hæmorrhage from the intestines occurred, and was followed by the discharge of pus, which, however, ceased when the patient took large quantities of lime water.

On the 28th of April, the patient had an enormous discharge from the intestines, which made him feel as if something had been torn out of his abdomen, and induced him to examine the evacuation. He found therein, in the midst of fæces and blood, a portion of ileum which measured two feet nine inches and a half along its convex border; it was dark and livid, but had a firm texture, and was still connected with a portion of mesentery. Its discharge was followed by a slight hæmorrhage and a considerable secretion of pus, which, however, again ceased after using lime-water. The patient was almost speechless with exhaustion, but the pain and the tumour had nearly disappeared. Tonics were administered, the fever diminished, and the strength rapidly increased. In the following October, he had regained all his former strength, and was hale and hearty, except that, on severe bodily exertion, the sensation of tension around the umbilicus, and of dragging of the stomach, would still return.—*Ib., from Hannoversche Annalen.*

---

*On the Presence of several Cysticerci in a Tumour having the appearance of a Boil.* By Dr. Fournier, of Craon.—A child, six years old, had a tumour of the size of a hen's egg on the superior and lateral part of the neck, which had only appeared four days. It was red, hot, painful, of a conical form, and circumscribed. On examining it with care, there appeared a small hole towards the base, in the middle of which a small white point was prominent, which had an almost imperceptible motion. A very limpid aqueous fluid flowed on pressure, and a particular but feeble sensation of *fremissement* was perceived. A species of clash (collision,) was distinguished by the ear; and the tumour, though red, hot, and painful, was soft and fluctuating, so that the presence of hydatids was diagnosticated. One of these worms was pressed out, and seven or eight more were removed by a small incision. They were afterwards recognized as cysticerci, having a very small roundish head, supported by a contracted neck. The body was formed of imbricated rings, perfectly visible to the naked eye; it was terminated by a small swelling, a kind of vesicle containing matter apparently albuminous. All performed some undulatory mo tions. The cure was complete on the seventh day.—*Ib., from Jour. des Conn. Med. Chirurg.*

